# Cannabidiol for Orofacial and Upper-Quarter Pain: A Systematic Evaluation of Therapeutic Potential

**DOI:** 10.3390/jcm14124186

**Published:** 2025-06-12

**Authors:** Karolina Walczyńska-Dragon, Jakub Fiegler-Rudol, Aleksandra Nitecka-Buchta, Stefan Baron

**Affiliations:** 1 Department of Temporomandibular Disorders, Medical University of Silesia in Katowice, 41-800 Zabrze, Poland; aleksandra.nitecka@sum.edu.pl (A.N.-B.); sbaron@sum.edu.pl (S.B.); 2 Student Scientific Society at the Department of Temporomandibular Disorders, Medical University of Silesia in Katowice, 41-800 Zabrze, Poland

**Keywords:** cannabidiol, upper-quarter disorders, temporomandibular disorders, orofacial pain, cannabinoid therapy

## Abstract

**Background:** Cannabidiol (CBD), a non-intoxicating phytocannabinoid, has garnered interest as a potential therapeutic agent for managing pain and inflammation associated with upper-quarter disorders, including temporomandibular disorders (TMDs), orofacial pain, myofascial dysfunction, and postoperative dental pain. **Methods**: This systematic review critically evaluated clinical, preclinical, and mechanistic studies on the efficacy and safety of CBD in the management of such conditions. A total of 10 studies, comprising randomized clinical trials and animal models, met the inclusion criteria and were assessed for methodological quality and risk of bias. **Results**: The findings suggest that CBD demonstrates analgesic, anti-inflammatory, and muscle-relaxant effects in chronic myofascial TMDs and bruxism, particularly when applied topically or intraorally. In contrast, studies on acute nociceptive pain (e.g., pulpitis, third molar surgery) yielded inconsistent results. Notably, CBD enhanced the effects of conventional analgesics such as opioids and NSAIDs in several preclinical models, indicating synergistic potential. However, substantial heterogeneity in CBD dosage, formulation, administration routes, and outcome measures limited comparability across studies. Adverse effects were minimal in clinical trials, yet underreported. **Conclusions**: While early evidence supports CBD’s potential as an adjunctive treatment for certain upper-quarter conditions, especially those involving chronic myofascial pain, further high-quality, condition-specific trials are required to establish standardized dosing, delivery methods, and long-term safety.

## 1. Introduction

Cannabidiol (CBD), a non-intoxicating phytocannabinoid primarily derived from Cannabis sativa, has garnered growing interest due to its potential analgesic, anti-inflammatory, and anxiolytic effects [1,2,3,4,5]. Although early research on CBD often focused on systemic conditions or generalized chronic pain [6,7], attention has increasingly turned to localized musculoskeletal and neuropathic disorders, including those of the so-called “upper quarter.” The upper quarter broadly encompasses the head, neck, shoulders, and upper back, an area in which persistent pain, inflammation, and functional deficits can significantly affect quality of life [8]. Temporomandibular disorders (TMDs) are a group of musculoskeletal and neuromuscular conditions involving the temporomandibular joint (TMJ), masticatory muscles, and associated structures. They are characterized by pain, restricted jaw movement, joint sounds, and functional limitations [9]. Within this region, temporomandibular disorders (TMDs), myofascial pain syndromes, dental/orofacial pathologies, and associated musculoskeletal conditions are common yet frequently challenging to manage with standard interventions [10,11]. Current frontline treatments for upper-quarter disorders typically consist of nonsteroidal anti-inflammatory drugs (NSAIDs), muscle relaxants, physical therapy, splint therapy, and, in more severe cases, opioid analgesics [12,13,14]. However, conventional pharmacological strategies can be accompanied by risks such as adverse systemic effects, drug tolerance, or dependency. Against this backdrop, CBD has emerged as a potentially safer, more targeted analgesic, demonstrating efficacy in modulating pain signals and reducing inflammatory responses via the endocannabinoid system [2,15]. Moreover, preclinical studies propose that CBD may enhance the effects of other analgesics, potentially allowing for lower doses of medications with higher side-effect profiles [16,17,18,19,20]. Despite promising indications, clinical data on its therapeutic capacity for upper-quarter disorders remain limited and sometimes contradictory [1,2,21,22]. Controversy also persists over the precise mechanisms by which CBD exerts its effects. While CBD appears to act indirectly on cannabinoid receptors, particularly CB_1 and CB_2, it may also influence non-cannabinoid receptor targets and modulate various enzymes involved in the metabolism of endocannabinoids [9,23,24,25,26,27,28]. This multifaceted pharmacology can complicate dosage determination, the route of administration, and outcome interpretation. Furthermore, differences in study design—including varying CBD formulations, concentrations, and adjunct therapies—have led to inconsistent clinical outcomes [4,29,30]. Given the growing popularity of CBD among patients seeking alternative or complementary treatments, a systematic examination of its evidence base in the context of upper-quarter disorders is timely and necessary. Previous reviews have explored the role of CBD in general pain management [3,31,32], yet few have specifically investigated how these effects translate to the unique physiological and biomechanical challenges posed by head, neck, shoulders, or orofacial pathologies. In addition, questions remain regarding its long-term safety, optimal delivery methods, and synergy with established interventions such as physical therapy or occlusal splinting [33]. In response to these gaps, the present systematic review was conducted to critically appraise and synthesize available data on the therapeutic potential of CBD for disorders of the upper quarter.

*Cannabis sativa* L., the plant from which cannabidiol (CBD) is derived, contains over 100 phytocannabinoids, of which tetrahydrocannabinol (THC) and CBD are the most studied. Unlike THC, CBD is non-intoxicating and interacts indirectly with the endocannabinoid system (ECS). It modulates ECS activity by inhibiting the degradation of endocannabinoids (such as anandamide) via FAAH inhibition and by interacting with non-cannabinoid targets including 5-HT1A, TRPV1, and PPAR-γ receptors [25,26,27]. These pathways underlie CBD’s anti-inflammatory, analgesic, and anxiolytic effects. Recent genomic and phytochemical analyses, including those by Mostafaei Dehnav et al. (2022) [34] and Balant et al. (2024) [35], underscore the biochemical diversity of *Cannabis sativa* L. and highlight how strain-specific cannabinoid profiles could influence therapeutic outcomes. Furthermore, emerging research advocates for the integration of cannabinoids into broader nutraceutical and health-focused formulations, as discussed by Salami et al. (2020), reinforcing CBD’s relevance beyond pharmacological contexts [36,37].

By collating evidence from animal models, in vitro studies, and clinical trials, we aimed to evaluate CBD’s effectiveness in pain relief, functional improvement, and overall patient-reported outcomes. Additionally, this review sought to highlight methodological strengths and limitations in existing research, provide insights into the risk of bias, and offer recommendations for future investigations. Through a rigorous assessment of the current data, we hope to contribute clarity to the ongoing discourse regarding the clinical utility of CBD and inform both practitioners and researchers of its potential role within the multimodal management of upper-quarter conditions.

## 2. Materials and Methods

### 2.1. Focused Question

A systematic review was conducted following the PICO framework, a structured method for formulating clinical research questions, as follows: In patients with disorders of the upper quarter (Population), does treatment with cannabidiol (Intervention), compared to standard pharmacological treatments, physical therapy, or placebo (Comparison), result in improved symptom relief, functional outcomes, or quality of life (Outcome)?

### 2.2. Search Strategy

**Figure 1:**
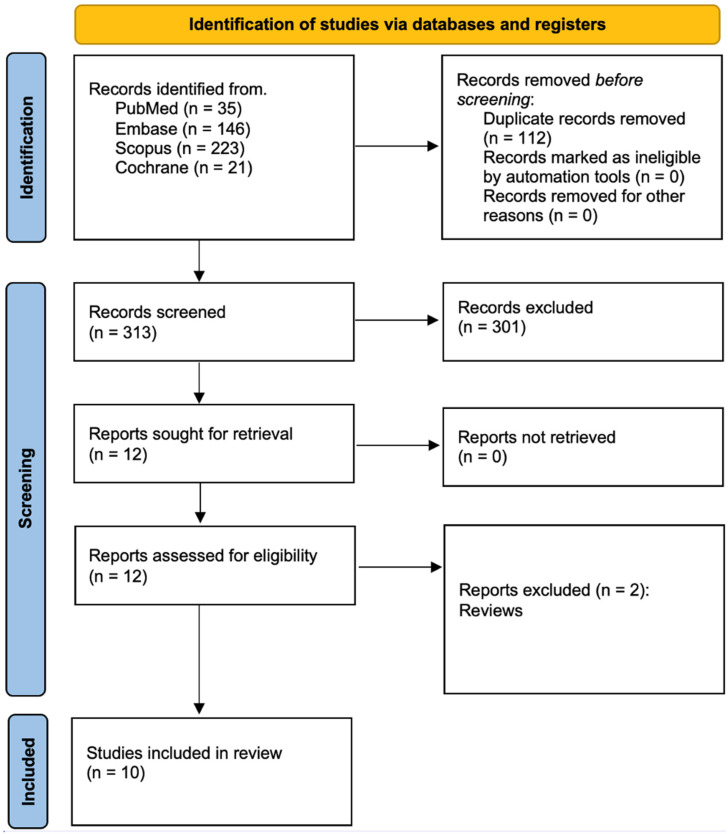
Prisma 2020 flow diagram.

This systematic review has been registered with PROSPERO under the assigned ID CRD420251009439. The study adhered to the Preferred Reporting Items for Systematic Reviews and Meta-Analyses (PRISMA 2020) guidelines [38]. The PRISMA (Preferred Reporting Items for Systematic Reviews and Meta-Analyses) guidelines are a set of evidence-based recommendations aimed at enhancing the transparency and completeness of reporting in systematic reviews. They provide a structured approach to literature identification, screening, inclusion, and synthesis. A comprehensive literature search was conducted across multiple electronic databases, including PubMed/Medline, Embase, Scopus, and the Cochrane Library, with detailed search strategies outlined in Figure 1. Three independent researchers performed the database searches using consistent search terms. Initial screening was based on titles and abstracts, ensuring alignment with predefined inclusion criteria (Table 1). Subsequently, two authors conducted a full-text review of the shortlisted studies to extract the relevant data. To further enhance the scope of the review, a snowball search was employed, examining the reference lists of eligible studies to identify additional relevant literature. This review aimed to evaluate the therapeutic potential of CBD in disorders of the upper quarter, assessing its efficacy in symptom relief, functional improvement, and overall quality of life compared to conventional treatments. Studies included in the final analysis were selected based on clearly defined inclusion and exclusion criteria.

### 2.3. Selection of Studies

To maintain an objective selection process in this systematic review, reviewers conducted independent evaluations of all retrieved study titles and abstracts. Any disagreements about eligibility were addressed through in-depth discussions until a mutual agreement was reached. This rigorous methodology, aligning with PRISMA guidelines, aimed to strengthen the review’s credibility by ensuring the inclusion of only the most pertinent and methodologically robust studies [38].

Inclusion Criteria:

- Studies investigating the therapeutic effects of CBD in disorders affecting the upper quarter (head, neck, shoulders, and upper back);
- Clinical, in vivo, and in vitro studies examining the impact of CBD on pain, inflammation, muscle function, or neurological disorders related to the upper quarter;
- Studies where CBD is used as the primary therapeutic agent for treating upper-quarter conditions;
- Research assessing the synergistic effects of CBD in combination with other pharmacological or non-pharmacological treatments for upper-quarter disorders;
- Controlled studies evaluating the effects of CBD compared to placebo, standard treatment, or alternative interventions for upper-quarter disorders;
- Comparative analyses examining the efficacy of CBD versus conventional treatment modalities for upper-quarter disorders;
- Longitudinal studies or those with follow-up periods assessing the sustained therapeutic effects of CBD in upper-quarter management.

Exclusion Criteria:

- Gray literature sources, case reports, letters to editors, narrative or systematic reviews, books, documents, and other non-journal materials;
- Non-peer-reviewed sources;
- Studies published in languages other than English;
- Duplicate studies or research sharing the same ethical approval number;
- General medical applications unrelated to disorders of the upper quarter;
- Studies without a control or comparison group;
- Research on CBD where it is not applied as a therapeutic intervention for upper-quarter disorders;
- Studies using cannabinoid compounds other than CBD;
- Studies focusing on conditions outside the upper quarter or those that do not specifically assess related disorders;
- In vitro studies that do not replicate physiological conditions relevant to upper-quarter disorders.

### 2.4. Risk of Bias in Individual Studies and Quality Assessment

During the preliminary stage of study selection, reviewers independently examined the titles and abstracts of the identified studies to minimize bias in the screening process. To assess the reliability of their evaluations, Cohen’s kappa statistic was utilized as an indicator of inter-reviewer agreement [39]. Any disagreements concerning the inclusion or exclusion of studies were addressed through in-depth discussions among the authors until a consensus was achieved.

The quality assessment of the seven included animal studies was conducted using the SYRCLE risk-of-bias tool, and the results are presented in Table 2 [39]. Across the studies, the risk of bias was generally low in domains such as baseline characteristics, incomplete outcome data, selective outcome reporting, and other sources of bias. However, most studies demonstrated an unclear risk of bias in key methodological domains, including sequence generation, allocation concealment, random housing, and the blinding of caregivers, investigators, and outcome assessors. This pattern suggests a lack of sufficient methodological detail in the reporting of experimental procedures, which limits the ability to fully appraise the internal validity of these studies. Despite these limitations, all studies received an overall SYRCLE judgment of low risk, indicating that no critical threats to validity were identified; however, caution is warranted due to the consistent presence of unclear risks in several domains.

The risk of bias was assessed for the three included randomized clinical trials using the RoB 2 tool, and the results are summarized in Table 3 [40]. All three studies demonstrated a low risk of bias across all five evaluated domains: the randomization process, deviations from intended interventions, missing outcome data, the measurement of the outcome, and the selection of the reported result. Consequently, each study was judged to have an overall low risk of bias, indicating high methodological quality and reducing the likelihood that bias influenced the reported findings.

**Table 2:** The results of the SYRCLE risk of bias tool across the animal studies.

Study	Sequence Generation	Baseline Characteristics	Allocation Concealment	Random Housing	Blinding (Caregivers/ Investigators)	Random Outcome Assessment	Blinding of Outcome Assessor	Incomplete Outcome Data	Selective Outcome Reporting	Other Sources of Bias	Overall SYRCLE Judgment
Ahn et al., 2007 [41]	Unclear risk	Low risk	Unclear risk	Unclear risk	Unclear risk	Unclear risk	Unclear risk	Low risk	Low risk	Low risk	Low risk
Brice-Tutt et al., 2025 [42]	Unclear risk	Low risk	Unclear risk	Unclear risk	Unclear risk	Unclear risk	Low risk	Low risk	Low risk	Low risk	Low risk
Burgos et al., 2010 [43]	Unclear risk	Low risk	Unclear risk	Unclear risk	Low risk	Unclear risk	Low risk	Low risk	Low risk	Low risk	Low risk
Laks et al., 2023 [44]	Unclear risk	Low risk	Unclear risk	Unclear risk	Low risk	Unclear risk	Low risk	Low risk	Low risk	Low risk	Low risk
Lee et al., 2008 [45]	Unclear risk	Low risk	Unclear risk	Unclear risk	Unclear risk	Unclear risk	Unclear risk	Low risk	Low risk	Low risk	Low risk
Wong et al., 2019 [46]	Unclear risk	Low risk	Unclear risk	Unclear risk	Low risk	Unclear risk	Low risk	Low risk	Low risk	Low risk	Low risk
Zubrzycki et al., 2017 [47]	Unclear risk	Low risk	Unclear risk	Unclear risk	Low risk	Unclear risk	Low risk	Low risk	Low risk	Low risk	Low risk

**Table 3:** The results of the RoB 2 tool across the RCTs.

Study	Randomization Process	Deviations from Intended Interventions	Missing Outcome Data	Measurement of the Outcome	Selection of the Reported Result	Overall RoB 2 Judgment
Nitecka-Buchta et al., 2019 [48]	Low risk	Low risk	Low risk	Low risk	Low risk	Low risk
Ostenfeld et al., 2011 [49]	Low risk	Low risk	Low risk	Low risk	Low risk	Low risk
Walczynska-Dragon et al., 2024 [50]	Low risk	Low risk	Low risk	Low risk	Low risk	Low risk

### 2.5. Data Extraction

Once an agreement was reached on the selected articles for inclusion, both authors systematically extracted relevant information to ensure consistency and completeness. The extracted data included the citation details (first author and publication year), study design, characteristics of the study population (sample size, age, and health condition), CBD dosage and administration route, composition of test and control groups, follow-up duration, and recorded outcomes related to upper-quarter disorders. Additionally, information regarding outcome measures (e.g., pain reduction, inflammation markers, functional improvement), statistical analyses used, and potential confounding factors was documented. Any discrepancies in data extraction were resolved through discussion to maintain accuracy and reliability.

## 3. Results

### 3.1. Study Selection

The study selection process, depicted in Figure 1, follows the PRISMA guidelines. Initially, 205 articles were retrieved through database searches. After eliminating duplicates, 313 unique records remained. A subsequent title and abstract screening removed unrelated studies. The 301 excluded studies were omitted based on predefined exclusion criteria, including a lack of control groups, the use of cannabinoid compounds other than CBD, the absence of relevance to upper-quarter disorders, publication in non-English languages, or failure to use CBD as a therapeutic intervention. As a result, 10 studies published within the past decade were included in the final analysis. The searches were conducted between the 2nd and 6th of March 2025. In total, 313 records were screened, but only 12 were determined to fit the criteria of this review.

### 3.2. Data Presentation

Table 4, Table 5 and Table 6 present a concise compilation of the information retrieved from the 12 studies that fulfilled the inclusion criteria and were incorporated into the review.

**Table 4:** A general overview of the studies.

Author and Year	Country	Study Design
Ahn et al., 2007 [41]	Republic of Korea, USA	Animal study
Brice-Tutt et al., 2025 [42]	USA	Animal study
Burgos et al., 2010 [43]	Spain	Animal study
Laks et al., 2023 [44]	USA	Animal study
Lee et al., 2008 [45]	Republic of Korea, USA	Animal study
Wong et al., 2019 [46]	Canada	Animal study
Zubrzycki et al., 2017 [47]	Germany, Poland	Animal study
Nitecka-Buchta et al., 2019 [48]	Poland	Randomized, double-blind clinical trial
Ostenfeld et al., 2011 [49]	UK, Italy, Germany	Randomized, controlled clinical trial
Walczynska Dragon et al., 2024 [50]	Poland	Randomized, double-blind clinical trial

**Table 5:** CBD parameters.

Study	CBD Administration Route	CBD Dosage/ Concentration	Type of Pain Studied	Study Model (Human/ Animal)	Additional Analgesics Used	Outcome Measures
Ahn et al., 2007 [41]	Intracisternal (direct injection into the cisterna magna)	10 µg, 30 µg, 80 µg WIN 55,212-2 (synthetic CB1/2 agonist)	Inflammatory temporomandibular joint pain	Animal (Sprague Dawley rats)	COX inhibitors: NS-398 (COX-2 inhibitor), Indomethacin (COX-1/2 inhibitor), Acetaminophen (COX-3 inhibitor), SC-560 (COX-1 inhibitor)	Number and duration of scratching behaviors in response to formalin-induced TMJ pain; Evans’ blue dye extravasation; motor function assessed via rotarod test
Brice-Tutt et al., 2025 [42]	Oral (gavage)	25 mg/kg	Orofacial cutaneous thermal pain	Animal (Sprague Dawley rats)	Oxycodone (1.4 mg/kg)	Lick/contact ratio in an operant orofacial pain assay; number of lick bursts; pain tolerance assessment
Burgos et al., 2010 [43]	Intraperitoneal injection	WIN 55,212-2 (0.5, 1 mg/kg, i.p.)	Orofacial inflammatory pain (formalin-induced)	Animal (rat)	Morphine (2, 5, 10 mg/kg, i.p.), Naloxone (1, 2 mg/kg, i.p.), Indomethacin (2.5, 5 mg/kg, i.p.), Ketamine (25, 50 mg/kg, i.p.)	Nociceptive behavior (face rubbing, head flinching), locomotor activity assessment
Laks et al., 2023 [44]	Intraperitoneal (i.p.)	5 mg/kg	Orofacial pain associated with pulpitis	Animal (Sprague Dawley rats)	None	CBD reduced AIF expression in trigeminal ganglia but did not significantly reduce orofacial sensitivity
Lee et al., 2008 [45]	Intracisternal injection	3, 10, or 30 μg	Temporomandibular joint nociception	Animal (Sprague Dawley rats)	Group II mGluR agonist (APDC) and Group III mGluR agonist (L-AP4)	30 μg WIN 55,212-2 significantly reduced scratching behavior by 75%; 3 μg WIN 55,212-2 enhanced the antinociceptive effects of APDC and L-AP4
Wong et al., 2019 [46]	Intramuscular injection	1 mg/mL and 5 mg/mL	Myofascial pain induced by nerve growth factor	Animal (female Sprague Dawley rats)	Cannabinol, Cannabichromene	CBD (5 mg/mL) significantly reduced NGF-induced mechanical sensitization. CBD/CBN combinations provided longer-lasting analgesia than either compound alone
Zubrzycki et al., 2017 [47]	Intracerebroventricular (i.c.v.) perfusion	50 nM and 100 nM	Orofacial pain induced by tooth pulp stimulation	Animal (male Long–Evans rats)	EM-2, URB597 (FAAH inhibitor), JZL195 (FAAH/MAGL dual inhibitor)	CBD significantly reduced pain response in a concentration-dependent manner. The antinociceptive effect was mediated by CB1 and μ receptors and enhanced by FAAH and FAAH/MAGL inhibitors
Nitecka-Buchta et al., 2019 [48]	Transdermal application	20% CBD oil formulation	Myofascial pain in masseter muscles	Human (patients with TMDs)	None	Significant reduction in masseter muscle sEMG activity (11% right, 12.6% left). VAS pain intensity reduced by 70.2% in CBD group compared to 9.81% in placebo group
Ostenfeld et al., 2011 [49]	Oral administration	100 mg and 800 mg	Acute postoperative pain after third molar extraction	Human (Patients undergoing third molar extraction)	Ibuprofen (800 mg pre-op + 400 mg post-op), Co-codamol (rescue analgesic)	GW842166 (100 mg and 800 mg) failed to provide significant analgesia compared to placebo. Ibuprofen was significantly more effective across all endpoints
Walczynska Dragon et al., 2024 [50]	Intraoral gel application	10% and 5% CBD gel	Muscle-related TMDs and sleep bruxism	Human (patients with TMDs and bruxism)	None	10% CBD gel reduced VAS pain by 57.4% and sEMG activity by 42.1%. 5% CBD gel reduced VAS pain by 40.8%. 10% CBD showed the highest bruxism index reduction (51%). Both CBD formulations were significantly more effective than placebo

TMJ, temporomandibular joint; WIN 55,212-2,Synthetic Cannabinoid Agonist; CB1, Cannabinoid Receptor Type 1; CB2, Cannabinoid Receptor Type 2; COX, Cyclooxygenase; NS-398, COX-2 Inhibitor; COX-2, Cyclooxygenase-2; Indomethacin, COX-1/2 Inhibitor; COX-1/2, Cyclooxygenase-1/2; Acetaminophen, COX-3 Inhibitor; COX-3, Cyclooxygenase-3; SC-560, COX-1 Inhibitor; COX-1, Cyclooxygenase-1; i.p., intraperitoneal; APDC, (2R,4R)-4-Aminopyrrolidine-2,4-dicarboxylate (Group II Metabotropic Glutamate Receptor Agonist); mGluR, Metabotropic Glutamate Receptor; L-AP4, L-2-Amino-4-phosphonobutyric acid (Group III Metabotropic Glutamate Receptor Agonist); NGF, Nerve Growth Factor; CBN, Cannabinol; CBC, Cannabichromene; i.c.v., Intracerebroventricular; nM, Nanomolar; EM-2, Endomorphin-2; URB597, Fatty Acid Amide Hydrolase (FAAH) Inhibitor; FAAH, Fatty Acid Amide Hydrolase; JZL195, Dual FAAH/Monoacylglycerol Lipase (MAGL) Inhibitor; MAGL, Monoacylglycerol Lipase; AEA, Anandamide; TMDs, temporomandibular disorders; sEMG, surface electromyography; VAS, Visual Analog Scale; pre-op, preoperatively; post-op, postoperatively; GW842166, Selective Cannabinoid Receptor Type 2 (CB2) Agonist.

**Table 6:** The main outcomes of each study.

Reference	Author and Year	Study Groups	Main Study Outcomes
[41]	Ahn et al., 2007	Formalin Control/Saline Control Groups: Formalin injected into TMJ (5% solution) + intracisternal saline or vehicle (DMSO/saline). Saline injected into TMJ (no formalin) to confirm no nociceptive response WIN 55,212-2 Groups: Formalin injection + low or high dose of WIN 55,212-2 (e.g., 10 μg or 30 μg, intracisternally) WIN 55,212-2 + Cannabinoid Antagonists Formalin injection + WIN 55,212-2 + CB1 antagonist (AM251) Formalin injection + WIN 55,212-2 + CB2 antagonist (AM630) WIN 55,212-2 + Naloxone: Formalin injection + WIN 55,212-2 + opioid receptor antagonist naloxone WIN 55,212-2 + COX Inhibitors: Formalin injection + sub-threshold WIN 55,212-2 (10 μg) + one of the following COX inhibitors: COX Inhibitors Alone: Formalin injection + one of the COX inhibitors (SC-560, NS-398, indomethacin, or acetaminophen) (without WIN 55,212-2) Dose–Response: WIN 55,212-2 ± NS-398: Formalin injection + increasing doses of WIN 55,212-2 (1, 3, 10, 30, or 80 μg). Formalin injection + NS-398 (COX-2 inhibitor) + same doses of WIN 55,212-2	Central activation of cannabinoid receptor CB1 significantly reduces nociception associated with TMJ inflammation, and blockade of central COX pathways, particularly COX-2 and putative COX-3, enhances this antinociceptive effect. Intracisternal administration of WIN 55,212-2, a non-selective CB1/2 agonist, effectively reduced formalin-induced TMJ pain in rats, with its analgesic effect blocked by CB1 antagonist AM251 but not by CB2 antagonist AM630, indicating that CB1 activation primarily mediates this effect. Furthermore, low doses of COX inhibitors alone did not alter nociception but significantly potentiated the effect of a sub-effective dose of WIN 55,212-2, suggesting that inhibition of central COX pathways augments cannabinoid-induced analgesia. Importantly, the study found no involvement of opioid pathways in this mechanism, as pretreatment with naloxone did not reverse cannabinoid-induced antinociception. These findings suggest that a combined administration of cannabinoids and COX inhibitors could offer a promising therapeutic strategy for managing inflammatory TMJ pain.
[42]	Brice-Tutt et al., 2025	Vehicle (Control): Rats received an oral gavage of the Cremophor–ethanol–water vehicle solution (no active drug). CBD Alone: Rats received cannabidiol 25 mg/kg by oral gavage. OXY Alone: Rats received oxycodone 1.4 mg/kg by oral gavage. CBD + OXY Combination: Rats received cannabidiol (25 mg/kg) and oxycodone (1.4 mg/kg) co-administered in a single oral gavage.	CBD, when co-administered with OXY, enhances opioid-induced analgesia in an orofacial thermal pain model using rats. This study is notable for its use of an oral administration route, aligning more closely with clinical applications. The results demonstrated that the combination of CBD and OXY provided greater pain relief than OXY alone, suggesting a potential role for CBD in reducing opioid dosages required for effective pain management. Additionally, the study observed that CBD alone exhibited analgesic-like properties, reinforcing prior preclinical findings. However, while preclinical data largely support the analgesic and opioid-sparing effects of CBD, clinical evidence remains inconsistent, highlighting the need for further research to confirm these benefits in human populations.
[43]	Burgos et al., 2010	Saline Control: Rats received saline (i.p.) prior to formalin injection. Cannabinoid Vehicle: Rats received the vehicle solution for WIN 55,212-2 (i.p.) prior to formalin. CB1 Antagonist Alone: Rats received SR141716A (CB1 antagonist) (i.p.) prior to formalin. CB2 Antagonist Alone: Rats received SR144528 (CB2 antagonist) (i.p.) prior to formalin. WIN 55,212-2: Rats received the cannabinoid agonist WIN 55,212-2 (0.5 or 1 mg/kg i.p.) prior to formalin. WIN 55,212-2 + CB1 or CB2 Antagonist: Rats received SR141716A or SR144528 (i.p.) 30 min before WIN 55,212-2, then formalin. Morphine: Rats received morphine (2, 5, or 10 mg/kg i.p.) prior to formalin. Morphine + Naloxone: Rats received naloxone (1 or 2 mg/kg i.p.) before morphine (5 or 10 mg/kg i.p.), then formalin. Indomethacin: Rats received indomethacin (2.5 or 5 mg/kg i.p.) prior to formalin. Ketamine: Rats received ketamine (25 or 50 mg/kg i.p.) prior to formalin.	WIN 55,212-2 significantly reduced nociceptive behavioral responses in inflammatory models of orofacial pain, including the TMJ formalin test, at doses of 0.5 and 1 mg/kg. The study found that this antinociceptive effect was mediated primarily by the CB1 receptor, as its action was blocked by the CB1-selective antagonist SR141716A but not by the CB2 antagonist SR144528. Additionally, WIN was as effective as morphine (10 mg/kg) and more effective than indomethacin and ketamine in attenuating inflammatory pain. Importantly, WIN-induced analgesia did not result in significant motor impairment, suggesting a strong therapeutic potential for cannabinoid-based treatments in managing upper-quarter inflammatory pain conditions.
[44]	Laks et al., 2023	Sham + Vehicle Underwent the sham procedure (no tooth pulp exposure) Treated systemically with a vehicle control solution Drilled (Pulp-Exposed) + Vehicle Underwent coronal pulpotomy in the left mandibular first molar (to create pulpitis). Treated systemically with the same vehicle control solutionDrilled (Pulp-Exposed) + CBDUnderwent coronal pulpotomy in the left mandibular first molar Treated systemically with 5 mg/kg of CBDDrilled (Pulp-Exposed) + β-Caryophyllene Underwent coronal pulpotomy in the left mandibular first molar Treated systemically with 30 mg/kg of β-CP	In this rat model of pulpitis, CBD at 5 mg/kg did not robustly reduce orofacial mechanical allodynia compared with vehicle treatment, but it did significantly decrease the expression of the microglial/macrophage marker AIF-1 in the ipsilateral trigeminal ganglion, suggesting a measurable anti-inflammatory effect. Although this reduction in AIF-1 did not translate into substantial behavioral analgesia—particularly when compared with β-caryophyllene, which more strongly attenuated both allodynia and inflammatory markers—the findings indicate that CBD can modulate aspects of neuroinflammation linked to dental orofacial pain, and therefore it warrants further investigation for its therapeutic potential in upper-quarter (orofacial) disorders.
[45]	Lee et al., 2008	Vehicle Control: Received the vehicle (e.g., saline or cyclodextrin solution) rather than active drugs Cannabinoid Agonist (WIN 55,212-2): Various doses (3, 10, or 30 μg) administered alone to assess dose-dependent effects on formalin-induced nociception Group II mGluR Agonist (APDC): Administered alone (multiple doses) to test antinociceptive effects against TMJ formalin injection Group III mGluR Agonist (L-AP4): Administered alone (multiple doses) similarly to assess formalin-induced nociception Combined Sub-Analgesic Cannabinoid + mGluR Agonist: Sub-threshold dose of WIN 55,212-2 (3 μg) given along with a sub-analgesic dose of APDC or L-AP4 to examine additive or synergistic antinociceptive effectsmGluR Antagonists: LY341495 (Group II antagonist) or CPPG (Group III antagonist) given before APDC or L-AP4, respectively, to confirm receptor-specific involvement	Activating central cannabinoid receptors markedly reduced inflammation-induced nociception in the TMJ and further revealed that sub-analgesic (i.e., low, non-impairing) doses of a cannabinoid agonist significantly enhanced the pain-relieving effects of metabotropic glutamate receptor (mGluR) agonists from groups II and III. Specifically, when the cannabinoid WIN 55,212-2 was administered together with mGluR agonists APDC (group II) or L-AP4 (group III) in a rodent model of TMJ pain, it both lowered the effective dose needed for relief and potentiated overall antinociceptive outcomes, without producing notable motor dysfunction. These findings underscore the therapeutic promise of combining cannabinoids with mGluR-based interventions for managing inflammatory pain in the TMJ—an approach that might extend to other upper-quarter disorders—by achieving strong analgesic effects at lower cannabinoid doses, thereby minimizing unwanted side effects.
[46]	Wong et al., 2019	Vehicle only (4% Tween 80 in saline) CBD at 1 mg/mL CBD at 5 mg/mL CBN at 1 mg/mL CBC at 1 mg/mL CBD + CBN (1:1) (1 mg/mL CBD + 1 mg/mL CBN) CBD + CBN (5:1) (5 mg/mL CBD + 1 mg/mL CBN)	In this rat model of NGF-induced masseter muscle pain, higher-dose cannabidiol (CBD at 5 mg/mL), as well as CBD alone and CBD/CBN combinations, effectively reversed mechanical sensitization without causing motor impairment or other central adverse effects. While CBC alone was not effective, combining CBD and CBN produced longer-lasting analgesia compared to either compound alone. These results suggest that non-psychoactive cannabinoids, particularly CBD and CBN, can act peripherally at the muscle to reduce pain in the upper quarter (e.g., as in temporomandibular disorders or fibromyalgia) and may therefore hold therapeutic potential for chronic muscle pain disorders with minimal central side effects.
[47]	Zubrzycki et al., 2017	Control- aCSF (vehicle), AEA AEA alone AEA + AM251 (CB₁ antagonist)/β-FNA (μ-antagonist) AEA + URB597 (FAAH inhibitor) AEA + JZL184 (MAGL inhibitor) AEA + JZL195 (FAAH/MAGL dual inhibitor) 2-AG 2-AG alone 2-AG + JZL184 (±AM251) EM-2 EM-2 alone EM-2 + β-FNA/AM251 EM-2 + URB597 EM-2 + AEA	In this study of orofacial pain using a rat model, the authors found that enhancing the endocannabinoid system—particularly by elevating levels of AEA through the inhibition of its degradative enzyme FAAH—produced significant antinociceptive effects. Although CBD was not directly tested, these results underscore that therapies targeting endocannabinoid signaling can suppress trigeminal nociceptive transmission in the brainstem, reduce pain responses, and work synergistically with opioid pathways. By extension, interventions that bolster or mimic the activity of endocannabinoids (including the use of phytocannabinoids like CBD) may offer promising, centrally mediated analgesic benefits for upper-quarter disorders such as orofacial pain syndromes, without some of the limitations often seen with traditional cannabinoid receptor agonists.
[48]	Nitecka-Buchta et al., 2019	Group 1 (Experimental/CBD Group): Received a topical formulation containing CBD Group 2 (Control/Placebo Group): Received a placebo topical formulation (no CBD)	Topical application of CBD significantly reduced both sEMG activity and perceived pain in masseter muscles of patients with myofascial pain, a common TMD. After two weeks of applying a CBD-based formulation, masseter muscle activity decreased by over 11% on average, and patients reported a 70% reduction in pain intensity, with no adverse effects. In comparison, those receiving a placebo formulation did not show statistically meaningful changes. These findings highlight CBD’s potential as a locally applied therapy for muscle relaxation, pain relief, and improvement in masticatory function, supporting broader investigations into CBD’s benefits for upper-quarter musculoskeletal disorders.
[49]	Ostenfeld et al., 2011	GW842166 (100 mg) + Placebo Received a single preoperative oral dose of GW842166 100 mg Received placebo at 4 h postoperatively GW842166 (800 mg) + Placebo Received a single preoperative oral dose of GW842166 800 mg Received placebo at 4 h postoperatively Ibuprofen Received an 800 mg ibuprofen dose preoperatively Received an additional 400 mg ibuprofen dose at 4 h postoperatively Placebo Received a placebo dose preoperatively Received a second placebo dose at 4 h postoperatively	In this randomized, double-blind, placebo-controlled study evaluating the analgesic efficacy of a selective cannabinoid receptor-2 agonist (GW842166) compared with ibuprofen or placebo in the context of acute pain after third molar extraction, the primary finding was that single oral doses of GW842166 (100 mg or 800 mg) failed to demonstrate clinically meaningful analgesia and showed little separation from the placebo across multiple pain outcomes, whereas ibuprofen produced significantly greater pain relief; moreover, no beneficial adjunctive effect emerged when GW842166 was coadministered with rescue analgesia, though the treatment was well tolerated from a safety standpoint, suggesting that under the conditions tested, the selective CB2 agonist did not yield therapeutic benefits in this acute postoperative pain model.
[50]	Walczynska-Dragon et al., 2024	Group 1a—Received a 10% CBD (cannabidiol) intraoral formulation Group 1b—Received a 5% CBD intraoral formulation Group 2 (Control)—Received a placebo intraoral formulation (no CBD)	The intraoral use of CBD formulations can significantly reduce pain, muscle tension, and sleep bruxism intensity in patients with TMDs, highlighting the therapeutic value of CBD for upper-quarter musculoskeletal conditions. In this randomized, double-blind clinical trial, patients receiving 10% CBD experienced the most substantial outcomes, with a 57.4% decrease in pain, a 42.1% reduction in muscle activity, and a 51% drop in sleep bruxism episodes, surpassing the improvements seen with the 5% CBD group. In contrast, the placebo group showed no meaningful changes in these measures. Overall, the findings indicate that higher-concentration CBD formulations exert a superior myorelaxant and analgesic effect, underscoring CBD’s promise as a non-invasive therapeutic option for disorders of the upper quarter involving muscular tension and pain.

TMJ, temporomandibular joint; DMSO, Dimethyl Sulfoxide; CB1, Cannabinoid Receptor Type 1; CB2, Cannabinoid Receptor Type 2; COX, Cyclooxygenase; NSAIDs, Non-Steroidal Anti-Inflammatory Drugs; CBD, Cannabidiol; OXY, Oxycodone; i.p., intraperitoneal; WIN 55,212-2, Synthetic Cannabinoid Agonist; APDC, (2R,4R)-4-Aminopyrrolidine-2,4-dicarboxylate (Group II mGluR Agonist), L-AP4—L-2-Amino-4-phosphonobutyric acid (Group III mGluR Agonist); LY341495, Group II mGluR Antagonist; CPPG, (RS)-α-Cyclopropyl-4-phosphonophenylglycine (Group III mGluR Antagonist); CBN, Cannabinol; CBC, Cannabichromene; NGF, Nerve Growth Factor; aCSF, Artificial Cerebrospinal Fluid; AEA, Anandamide; FAAH, Fatty Acid Amide Hydrolase; MAGL, Monoacylglycerol Lipase; 2-AG, 2-Arachidonoylglycerol; EM-2, Endomorphin-2; β-FNA, Beta-Funaltrexamine (μ-Opioid Receptor Antagonist); JZL184—MAGL Inhibitor; JZL195, Dual FAAH/MAGL Inhibitor; GW842166, Selective CB2 Agonist; sEMG, surface electromyography.

## 4. Discussion

### 4.1. Results in the Context of Other Evidence

CBD demonstrates promising therapeutic potential for managing upper-quarter disorders, particularly in conditions involving orofacial pain and inflammation. In temporomandibular joint (TMJ) inflammation models, CBD significantly reduces nociceptive responses, with enhanced effectiveness observed when combined with cyclooxygenase inhibitors. This suggests that a combination of CBD and conventional anti-inflammatory medications could yield synergistic therapeutic outcomes [41]. Furthermore, combining CBD with opioids such as oxycodone substantially improves analgesic efficacy in orofacial pain models. This combination therapy may allow clinicians to lower opioid dosages, potentially reducing associated adverse effects while maintaining effective pain management [42]. Mechanistically, the activation of cannabinoid receptors, particularly CB1, robustly attenuates inflammatory pain in TMJ disorders.

The included studies evaluated a spectrum of conditions, ranging from acute inflammatory pulpitis and nociception induced by tooth pulp stimulation to chronic myofascial pain associated with temporomandibular disorders (TMDs). These conditions differ markedly in their underlying pathophysiology. For instance, pulpitis involves the acute, localized inflammation of the dental pulp, characterized primarily by peripheral nociceptive sensitization, whereas chronic TMD pain often reflects a combination of peripheral muscle hyperactivity, central sensitization, and psychosocial factors. As such, findings from acute orofacial pain models cannot be directly extrapolated to chronic, multifactorial conditions like TMDs. This heterogeneity limits the comparability of study outcomes and highlights the need for disorder-specific investigations when evaluating the therapeutic effects of CBD.

While the therapeutic effects of CBD—particularly in pain relief and inflammation—have been reported in prior studies, the novelty of this systematic review lies in its focused synthesis of CBD’s applications specifically within the context of upper-quarter disorders, a domain characterized by complex musculoskeletal and neuropathic overlap. Unlike general reviews on chronic pain or systemic cannabinoid use, this review integrates clinical, animal, and mechanistic data across a well-defined anatomical region (head, neck, shoulders, and upper back), encompassing conditions such as TMDs, orofacial pain, and myofascial dysfunction. By highlighting the heterogeneity in formulations, dosages, and outcome measures, this work identifies critical gaps in the existing literature and presents a nuanced interpretation that is both disorder-specific and mechanistically informed. To our knowledge, no previous review has comprehensively mapped the potential of CBD across this anatomically and clinically distinct group of disorders.

Although cannabidiol (CBD) exhibits a relatively low binding affinity for classical cannabinoid receptors CB_1_ and CB_2_, its modulatory effects on pain pathways extend beyond these targets, thereby contributing to its analgesic potential and its capacity to enhance the efficacy of other analgesics. One key mechanism involves the inhibition of fatty acid amide hydrolase (FAAH), the enzyme responsible for degrading anandamide, an endogenous cannabinoid. By preserving anandamide levels, CBD indirectly augments CB₁-mediated signaling, which plays a crucial role in nociceptive modulation. Furthermore, CBD has been shown to act on several non-cannabinoid targets, including the serotonin 5-HT1A receptor, transient receptor potential vanilloid type 1 (TRPV1) channels, and peroxisome proliferator-activated receptor gamma (PPAR-γ). These interactions are believed to contribute to anxiolytic, anti-inflammatory, and antinociceptive effects [25,26,27,28]. Notably, through the modulation of TRPV1 and adenosine uptake inhibition, CBD can influence neuronal excitability and inflammatory cascades, mechanisms that may underlie its observed potentiation of opioids and NSAIDs in preclinical models [42,43]. These multimodal pathways highlight the pharmacodynamic versatility of CBD and suggest that its therapeutic value may lie as much in its ability to enhance the action of other agents as in its own direct effects.

Notably, the analgesic effects mediated via CB1 activation were comparable to morphine and superior to many common analgesics, indicating that cannabinoid modulation represents a potent therapeutic target for inflammatory orofacial conditions [43]. However, specific dosage considerations are critical. For example, CBD administered at a dose of 5 mg/kg significantly diminished inflammation in dental pulpitis but exhibited limited direct analgesic properties, suggesting that its primary mode of action might be anti-inflammatory rather than acute pain relief [44]. Additionally, cannabinoids at sub-analgesic doses potentiate the effects of metabotropic glutamate receptor (mGluR) agonists, highlighting potential synergistic therapeutic strategies for managing TMJ inflammation through combined cannabinoid and receptor-specific therapies [45]. The peripheral administration of CBD has also demonstrated efficacy by significantly reducing mechanical sensitization associated with muscle-related orofacial pain. This peripheral route could potentially provide analgesic benefits with a reduced risk of central side effects, enhancing therapeutic safety profiles for chronic pain management [46]. Conversely, the central modulation of the endocannabinoid system in the brainstem also effectively reduces centrally mediated orofacial pain, underscoring the importance of targeting central cannabinoid mechanisms in certain chronic pain conditions [47]. Topical CBD applications further expand therapeutic options, significantly reducing pain and muscle activity in patients suffering from temporomandibular disorders (TMDs). This topical route suggests CBD’s potential as a localized, effective treatment in specifically targeting myofascial pain [48]. Interestingly, selective CB2 receptor agonists did not produce meaningful analgesia in acute postoperative dental pain, suggesting that receptor-selective strategies alone might be insufficient for acute pain management and highlighting the complexity of cannabinoid analgesic mechanisms [49]. In contrast, intraoral CBD gel, especially at higher concentrations (10%), effectively reduced pain intensity, muscle tension, and bruxism episodes, reinforcing CBD’s therapeutic versatility in managing muscular and functional disorders within the upper-quarter region [50]. However, findings from clinical research have presented mixed results regarding cannabinoid effectiveness for orofacial pain. For instance, Votrubec et al. reported limited clinical evidence, noting significant analgesia in only one of five reviewed studies evaluating topical cannabidiol use for TMD-related pain [51]. Similarly, Côté et al. observed that the oral administration of synthetic cannabinoid nabilone did not significantly relieve pain compared to a placebo among head and neck cancer patients undergoing radiotherapy, further challenging the consistent efficacy of cannabinoids for acute orofacial pain [52]. Kalliomäki et al. also found no substantial analgesic benefit of the synthetic cannabinoid AZD1940 over a placebo in patients undergoing the surgical extraction of impacted third molars, emphasizing the variability in cannabinoid efficacy across acute pain scenarios [53]. Consistent with these findings, Raft et al. reported negligible analgesic effects following intravenous THC administration, with several participants even expressing a preference for the placebo, highlighting patient-specific variability in cannabinoid responsiveness [54,55,56,57,58,59,60]. Conversely, numerous studies provide compelling evidence supporting cannabinoids’ efficacy for chronic neuropathic pain. Lynch and Ware noted significant analgesic outcomes in seven of eleven high-quality trials examining cannabinoids, with notable improvements in secondary measures including sleep quality, muscle stiffness, and spasticity. The observed adverse effects were generally mild to moderate and well tolerated, suggesting a favorable therapeutic profile in chronic conditions [61,62,63,64,65,66,67,68]. Similarly, Aviram et al. concluded that cannabis-based medicines show modest but statistically significant efficacy in managing chronic neuropathic pain. Notably, inhalation methods appeared particularly effective, although the clinical significance of these outcomes remains uncertain, and gastrointestinal side effects appeared more frequently with oral formulations [69,70,71,72,73,74]. Allan et al. provided a more conservative interpretation, suggesting that while medical cannabinoids might moderately alleviate nausea, vomiting, and spasticity, the evidence for significant analgesic benefits in chronic neuropathic pain remains limited. Frequent and common adverse effects further complicate the assessment of the overall clinical benefit [75,76,77]. Nonetheless, Wong et al. demonstrated effective myofascial pain reduction through intramuscular CBD administration, indicating the potential for peripheral analgesic application and supporting a broader therapeutic scope for cannabinoids in chronic pain management [78]. Among the three randomized clinical trials included [48,49,50], adverse effects were either minimal or not reported. Nitecka-Buchta et al. [48] and Walczyńska-Dragon et al. [50] observed no adverse events in patients receiving topical or intraoral CBD formulations, suggesting good tolerability. In contrast, Ostenfeld et al. [49] noted that while the CB2 agonist GW842166 was well tolerated, it did not produce significant analgesia, and no CBD-specific adverse effects were reported. However, the absence of detailed adverse event reporting in some studies limits definitive conclusions regarding safety.

### 4.2. Limitations of the Evidence

Despite the promising findings regarding the therapeutic potential of CBD for disorders affecting the upper quarter (head, neck, shoulders, and upper back), several limitations must be acknowledged. First, study heterogeneity remains a significant challenge. The reviewed studies vary widely in terms of CBD formulations, dosages, administration routes, and treatment durations, making direct comparisons difficult. While some studies investigated topical CBD application for myofascial pain, others utilized systemic administration (e.g., oral or intraperitoneal routes), which may lead to differing pharmacokinetic profiles and therapeutic effects. The variability in treatment protocols complicates the ability to draw definitive conclusions about the most effective mode of CBD administration for upper-quarter disorders. Second, the methodological quality varied across studies, with notable differences in sample sizes, control conditions, and outcome measures. While randomized controlled trials were prioritized, some studies lacked proper blinding, allocation concealment, or objective pain assessments, increasing the risk of bias. Additionally, small sample sizes in certain clinical trials limit the statistical power and reduce the generalizability of the findings to broader patient populations. Third, the short follow-up periods in many studies pose a limitation in assessing the long-term efficacy and safety of CBD. Given that disorders of the upper quarter—such as temporomandibular disorders, myofascial pain, and bruxism—often present as chronic conditions, extended observation periods are necessary to evaluate sustained benefits, potential tolerance development, and delayed adverse effects. Another key concern is publication bias, where studies reporting positive outcomes are more likely to be published, potentially skewing the evidence base. The absence of large-scale, multi-center trials examining CBD’s impact on upper-quarter conditions further limits the robustness of the conclusions drawn from this review. Finally, a lack of standardized outcome measures remains a critical issue. Studies employed different tools to assess pain (e.g., Visual Analog Scale, pressure pain threshold, sEMG), making it difficult to perform direct comparisons or meta-analyses. The inconsistent use of validated psychometric tools hinders reproducibility and limits the clinical applicability of the findings. Another limitation is related to the generalizability of the findings across different study models. While animal and in vitro studies provide valuable mechanistic insights—particularly regarding receptor-level activity and anti-inflammatory responses—their translational relevance to human pathology is inherently constrained. Differences in dosing, metabolism, and behavioral endpoints complicate the extrapolation of preclinical findings to clinical practice. Moreover, in vitro models often fail to replicate the complex tissue and neuroimmune interactions observed in vivo. Although the inclusion of diverse study types strengthens the theoretical foundation for CBD use, future studies must bridge these translational gaps through robust clinical trials that validate preclinical hypotheses in real-world patient populations. Additionally, the reporting of adverse effects was inconsistent across the included clinical studies. While most trials described CBD as well tolerated, the lack of standardized adverse event reporting restricts the ability to fully evaluate its safety profile. Future trials should incorporate the systematic documentation of side effects using validated criteria.

All three randomized clinical trials included in this review [48,49,50] involved placebo-controlled designs. In the study by Nitecka-Buchta et al. [48], topical CBD application led to a 70.2% reduction in masseter pain intensity compared to a 9.8% reduction in the placebo group, alongside significant decreases in sEMG activity. Walczyńska-Dragon et al. [50] similarly reported that both 5% and 10% intraoral CBD formulations outperformed the placebo, with the 10% gel yielding a 57.4% reduction in pain and a 51% decrease in sleep bruxism frequency, compared to negligible changes in the placebo group. In contrast, Ostenfeld et al. [49] found no statistically significant difference between the CBD-derived CB2 agonist (GW842166) and the placebo in managing acute postoperative dental pain, with ibuprofen performing markedly better than both. These results indicate that while CBD shows strong efficacy compared to placebos in chronic myofascial TMD-related pain, it appears less effective in acute nociceptive pain contexts.

### 4.3. Limitations of the Review Process

One major limitation of this systematic review is the considerable heterogeneity across the included studies. Variations in study design, CBD formulations, dosages, administration routes, and outcome measures make it challenging to directly compare findings and draw definitive conclusions about the therapeutic potential of CBD for upper-quarter disorders. Additionally, much of the evidence stems from preclinical animal models, which limits the immediate applicability of the results to human clinical settings. The review also restricted its search to studies published in English, potentially introducing language bias and overlooking relevant research in other languages. Furthermore, inconsistencies in methodological quality and risk of bias among the included studies may have affected the reliability of the synthesized evidence, underscoring the need for more standardized and robust clinical trials in the future. Another notable limitation is the potential for publication bias. By restricting inclusion to peer-reviewed journal articles, the review aimed to ensure methodological rigor and data reliability. However, this criterion may inadvertently skew the evidence base, as studies reporting positive or statistically significant outcomes are more likely to be accepted for publication. As a result, relevant findings with null or negative results, which are less frequently published, may have been excluded, potentially overestimating the therapeutic efficacy of CBD in upper-quarter disorders.

### 4.4. Implications for Practice, Policy, and Future Research

The findings of this review suggest that while CBD shows promise as a therapeutic option for upper-quarter disorders, its current application in clinical practice remains provisional. For practitioners, these results highlight the potential benefits of integrating CBD, particularly in cases where conventional therapies have limitations, but they also underscore the importance of individualized treatment plans due to variations in dosage, formulation, and administration routes. From a policy perspective, there is a clear need for regulatory frameworks that ensure product consistency, safety, and efficacy, which in turn would facilitate more standardized clinical guidelines and insurance coverage. Future research must prioritize large-scale, well-designed clinical trials that address existing methodological gaps, as well as exploring long-term safety outcomes and potential interactions with other treatments. Collectively, these efforts will be critical in translating preliminary evidence into reliable, evidence-based practices that can effectively guide patient care and inform public health policies.

While the current review acknowledges heterogeneity in CBD formulations, dosages, and administration routes, further synthesis reveals emerging trends that could inform a preliminary dose–response framework. For instance, clinical studies employing higher-concentration topical or intraoral formulations (e.g., 10% CBD gel or oil) demonstrated superior efficacy in reducing pain and muscle activity compared to lower-concentration or systemic applications [48,50]. Similarly, preclinical models suggest a dose-dependent relationship, with sub-effective doses potentiating other analgesics and higher doses achieving direct antinociceptive effects [41,45]. However, dose escalation is not uniformly associated with improved outcomes, and the therapeutic window appears to vary by delivery route and target condition. This underscores the need for future research to delineate optimal dosage thresholds and pharmacokinetic profiles tailored to specific upper-quarter pathologies.

To advance clinical translation, future research should prioritize specific upper-quarter conditions where current therapies show limited efficacy—such as chronic temporomandibular disorders, myofascial pain, and sleep-related bruxism. Comparative effectiveness trials evaluating CBD against standard treatments (e.g., NSAIDs, muscle relaxants, splint therapy) are urgently needed, ideally using randomized, double-blind designs and standardized outcome measures. Moreover, investigations should explore the relative effectiveness of distinct CBD formulations (topical, transdermal, intraoral, or oral) and dosing regimens, including synergistic combinations with conventional pharmacological or physiotherapeutic interventions. Longitudinal trials assessing sustainability of effects, safety, and potential for tolerance are also crucial. A coordinated effort toward protocol harmonization will enhance cross-study comparability and facilitate evidence-based guidelines for CBD use in upper-quarter disorders.

## 5. Conclusions

This systematic review synthesizes evidence on the potential therapeutic effects of cannabidiol (CBD) across a range of upper-quarter disorders, including temporomandibular disorders (TMDs), pulpitis, myofascial pain, and acute postoperative pain. While CBD demonstrated analgesic, anti-inflammatory, and muscle-relaxant effects in several preclinical and clinical models, the diversity in pain etiologies, study designs, and outcome measures precludes direct extrapolation to any single condition, including TMDs. Clinical trials evaluating topical or intraoral CBD showed promising results in patients with myofascial TMD-related pain and bruxism, whereas studies on acute nociceptive pain—such as postoperative or pulp-induced pain—produced mixed or inconclusive outcomes. Given these variations, current evidence supports cautious optimism regarding CBD’s role in managing select upper-quarter pain conditions but not as a generalized treatment for TMDs. Rigorous, condition-specific trials with standardized protocols are essential to establish the efficacy, safety, and optimal use of CBD across distinct orofacial and musculoskeletal disorders.

## Figures and Tables

**Table 1:** Search syntax used in the study.

Source	Search Term	Number of Results
PubMed	((Cannabidiol [MeSH Terms] OR CBD[TIAB] OR “cannabidiol”[TIAB] OR “medical cannabis”[TIAB] OR “Medical Marijuana”[MeSH Terms] OR “cannabinoids”[MeSH Terms] OR “cannabinoid”[TIAB]) AND (Temporomandibular Joint Disorders[MeSH Terms] OR TMD[TIAB] OR “temporomandibular disorder”[TIAB] OR “TMJ disorder”[TIAB] OR “orofacial pain”[TIAB] OR “Facial Pain”[MeSH Terms] OR “jaw pain”[TIAB] OR “myofascial pain”[TIAB] OR “Myofascial Pain Syndromes”[MeSH Terms]))	35
Embase	(‘cannabidiol’/exp OR ‘cbd’:ti,ab OR ‘cannabidiol’:ti,ab OR ‘medical cannabis’:ti,ab OR ‘medical marijuana’/exp OR ‘medical marijuana’:ti,ab OR ‘cannabinoids’/exp OR ‘cannabinoid’:ti,ab) AND (‘temporomandibular joint disorder’/exp OR ‘tmd’:ti,ab OR ‘temporomandibular disorder’:ti,ab OR ‘tmj disorder’:ti,ab OR ‘orofacial pain’:ti,ab OR ‘facial pain’/exp OR ‘facial pain’:ti,ab OR ‘jaw pain’:ti,ab OR ‘myofascial pain’:ti,ab OR ‘myofascial pain syndromes’/exp OR ‘myofascial pain syndromes’:ti,ab)	146
Scopus	(TITLE-ABS-KEY(cannabidiol) OR TITLE-ABS-KEY(CBD) OR TITLE-ABS-KEY(“medical cannabis”) OR TITLE-ABS-KEY(“medical marijuana”) OR TITLE-ABS-KEY(cannabinoids) OR TITLE-ABS-KEY(cannabinoid)) AND (TITLE-ABS-KEY(“Temporomandibular Joint Disorders”) OR TITLE-ABS-KEY(TMD) OR TITLE-ABS-KEY(“temporomandibular disorder”) OR TITLE-ABS-KEY(“TMJ disorder”) OR TITLE-ABS-KEY(“orofacial pain”) OR TITLE-ABS-KEY(“Facial Pain”) OR TITLE-ABS-KEY(“jaw pain”) OR TITLE-ABS-KEY(“myofascial pain”) OR TITLE-ABS-KEY(“Myofascial Pain Syndromes”))	223
Cochrane	((MH “Cannabidiol” OR “Cannabidiol”) OR (MH “CBD” OR “CBD”) OR (MH “Medical Cannabis” OR “Medical Cannabis”) OR (MH “Medical Marijuana” OR “Medical Marijuana”) OR (MH “Cannabinoids” OR “Cannabinoids”) OR (MH “Cannabinoid” OR “Cannabinoid”)) AND ((MH “Temporomandibular Joint Disorders” OR “Temporomandibular Joint Disorders”) OR (MH “TMD” OR “TMD”) OR (MH “Temporomandibular Disorder” OR “Temporomandibular Disorder”) OR (MH “TMJ Disorder” OR “TMJ Disorder”) OR (MH “Orofacial Pain” OR “Orofacial Pain”) OR (MH “Facial Pain” OR “Facial Pain”) OR (MH “Jaw Pain” OR “Jaw Pain”) OR (MH “Myofascial Pain” OR “Myofascial Pain”) OR (MH “Myofascial Pain Syndromes” OR “Myofascial Pain Syndromes”))	21

## Data Availability

Not applicable.
